# Cytomegalovirus-Associated Central Pontine Myelinolysis in a Severely Immunocompromised HIV/AIDS Patient

**DOI:** 10.7759/cureus.98376

**Published:** 2025-12-03

**Authors:** Harvey B De Nieva, Wongelawit Zerihun, Egesh Aryal, Dhiraj Nalabothu, Laxman Wagle

**Affiliations:** 1 Internal Medicine, Ascension Saint Agnes Hospital, Baltimore, USA; 2 Internal Medicine, Nepalese Army Institute of Health Sciences, Kathmandu, NPL; 3 Internal Medicine, Trinity Medical Sciences University, Kingstown, VCT

**Keywords:** aids, cpm, hiv, ods, radiology

## Abstract

Central pontine myelinolysis (CPM) is most commonly associated with rapid correction of hyponatremia, yet emerging evidence indicates that additional factors, particularly in immunocompromised individuals, may also play a role. We report a case of a 35-year-old male with untreated HIV/AIDS and stage IV Hodgkin lymphoma undergoing chemotherapy, who presented with acute hypoxic respiratory failure. Despite no fluctuations in serum sodium and normal serum osmolarity and electrolytes, MRI revealed a classic trident-shaped lesion in the central pons consistent with CPM. Extensive workup showed a markedly elevated cytomegalovirus (CMV) viral load, while cerebrospinal fluid findings were inconclusive. In the context of profound immunosuppression and absence of other identifiable causes, CMV-associated CPM was strongly suspected. Initiation of antiviral therapy led to partial neurological improvement, but the patient ultimately left the hospital against medical advice. This report underscores the importance of considering opportunistic infections such as CMV as potential contributors to CPM in patients with advanced HIV, even in the absence of classic metabolic triggers. Broader awareness of these atypical etiologies may facilitate earlier recognition and more targeted management in this vulnerable population.

## Introduction

Central pontine myelinolysis (CPM), a subset of osmotic demyelination syndrome (ODS), is a rare demyelinating disorder of the pons [1]. Initially associated with chronic alcoholism and malnutrition, the understanding of CPM pathogenesis expanded in the early 1980s with the recognition that rapid correction of hyponatremia is a key precipitating factor [2]. The clinical spectrum of CPM varies significantly, ranging from asymptomatic cases to severe manifestations such as locked-in syndrome, coma, or death [3]. While CPM is most commonly associated with sodium disturbances, reports implicating infections as a causative factor remain exceedingly rare. In adults, only a handful of cases have linked CPM to HIV/AIDS in the presence of minimal hyponatremia. For example, one case involved an AIDS patient with slowly corrected mild hyponatremia and hypoalbuminemia, who developed CPM, prompting hypotheses that immune dysregulation, low albumin, or opportunistic disease burden may increase vulnerability to osmotic stress [4]. In this report, we present a rare case of putative CMV-associated CPM in an adult patient with untreated HIV/AIDS.

## Case presentation

The patient was a 35-year-old male with a significant past medical history of an untreated HIV/AIDS and a recently diagnosed stage IV classic Hodgkin lymphoma. His HIV had progressed to an advanced stage, with a CD4 count of nil, reflecting profound immunosuppression due to noncompliance with antiretroviral therapy. His wife reported that he had been compliant with bictegravir-emtricitabine-tenofovir alafenamide since January 2025 under the supervision of his primary care physician, and she denied any recent alcohol use. He was under the care of an oncologist at a tertiary hospital and had recently initiated treatment with the brentuximab vedotin, doxorubicin, vinblastine, and dacarbazine (BV-AVD) regimen for his lymphoma, and had recently received the first cycle of chemotherapy three days before admission, further compromising his already weakened immune system. He had also been scheduled to see an HIV specialist for re-initiation of antiretroviral therapy, but had not done so before this admission.

The patient presented to the emergency department with acute shortness of breath that had been progressively worsening. On arrival, he was in severe respiratory distress and tachypneic, with oxygen saturation of 70% on room air. Other vital signs included a temperature of 37.6 °C, blood pressure of 120/80 mmHg, and a BMI of 19.7 kg/m². He was also obtunded and required immediate intubation. A full neurologic examination could not be performed due to sedation (RASS goal -3 to -4) with propofol and dexmedetomidine infusions. Laboratory evaluation revealed alarming findings consistent with his severe immunocompromised state. His absolute neutrophil count was 252 k/uL (normal range: 2,500-6,000 k/µL), and his CD4 count was 0 cells/mm³ (normal: >500 cells/mm³). Also, his complete blood count showed thrombocytopenia, while his chemistry panel was mostly unremarkable with normal serum electrolyte, glucose, renal function, and liver enzyme levels except for normal anion gap metabolic acidosis, hypoalbuminemia, and elevated lactate dehydrogenase (Table 1).

**Table 1:** Initial laboratory test results on admission ESR: erythrocyte sedimentation rate; AST: aspartate aminotransferase; ALT: alanine aminotransferase; TSH: thyroid-stimulating hormone

Variable	Result	Normal values
White blood count	6.9 k/uL	4.0-11.0 K/uL
Hemoglobin	6.9 g/dL	13.0-17.0 g/dL
Hematocrit	21.1%	41.0-50.0%
Platelet count	111 K/uL	150-400 K/uL
Neutrophils	90%	35.0-70.0%
Band	1%	0-5%
Lymphocyte	5%	20-45%
Monocyte	3%	4.0-12.5%
Atypical lymphocyte	1%	0-5%
ESR	>145 mm/hr	0-5 mm/hr
Serum sodium	137 meq/L	136-145 meq/L
Serum potassium	4.1 meq/L	3.5-5.1 meq/L
Serum chloride	106 meq/L	98-107 meq/L
Serum carbon dioxide	19.0	22-29 meq/L
Anion gap	12 meq/L	9-18 meq/L
Blood urea nitrogen	22 mg/dL	8.9-20.6 mg/dL
Serum creatinine	0.8 mg/dL	0.72-1.25 mg/dL
Serum calcium	8.8 mg/dL	8.4-10.2 mg/dL
Serum phosphorus	3.5 mg/dL	2.3-4.7 mg/dL
Serum magnesium	2.2 mg/dL	1.6-2.6 mg/dL
Random blood glucose	156 mg/dL	70-100 mg/dL
Serum total bilirubin	0.7 mg/dL	0.2-1.2 mg/dL
Serum AST	32 U/L	5-34 U/L
Serum ALT	47 U/L	0-55 U /L
Serum lactate dehydrogenase	424 U/L	125-220 U/L
Serum total protein	6.9 gm/dL	6.4-8.3 gm/dL
Serum albumin	2.8 gm/dL	3.5-5.2 gm/dL
Serum globulin	3.6 gm/dL	-
TSH	0.36 mIU/L	0.35-4.94 mIU/L
Absolute lymphocyte	0.0 x10^3^/uL	0.7-3.1 x10^3^/uL

**Figure 1:**
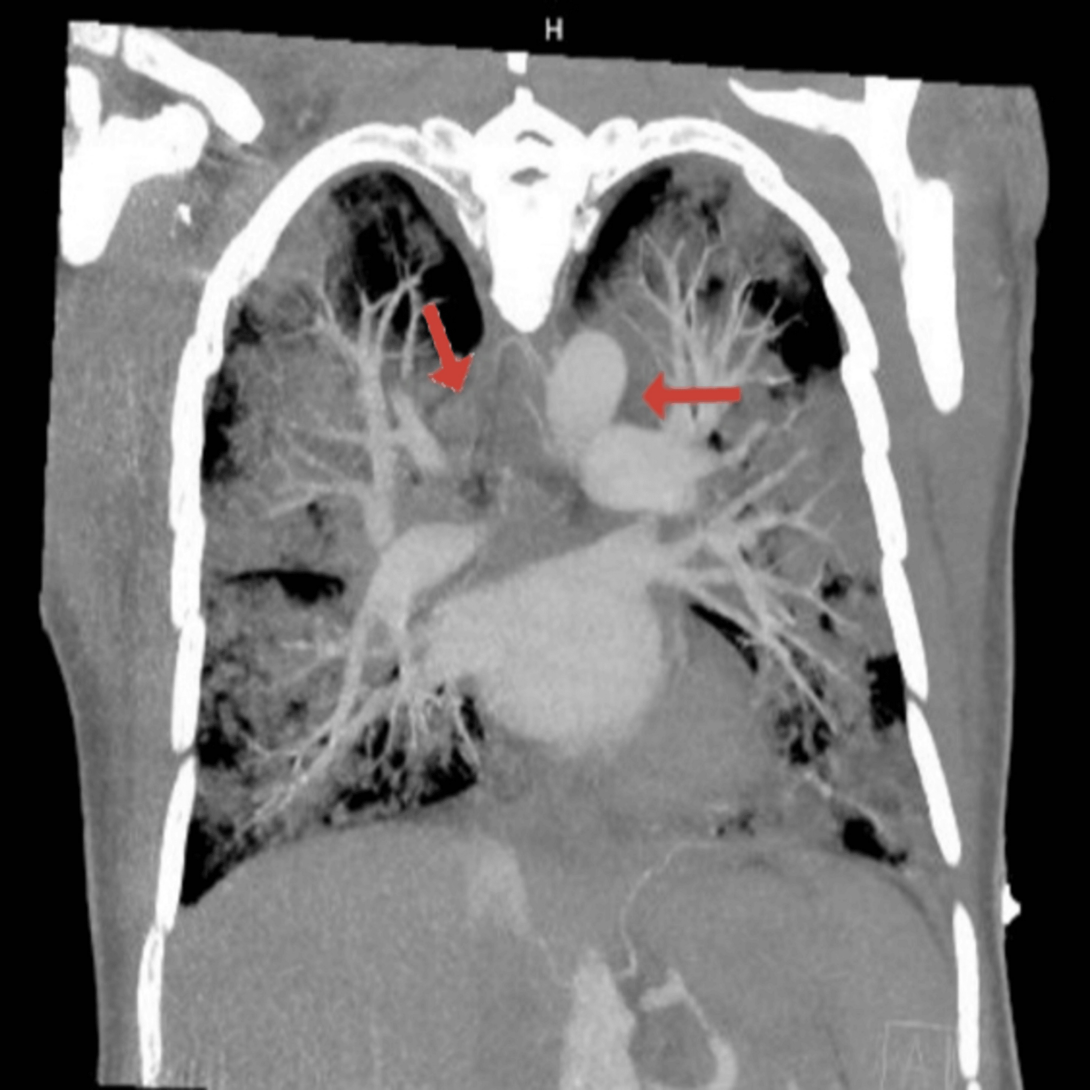
CT chest with angiography The image shows bulky mediastinal, prevascular, and axillary adenopathy; diffuse moderate to severe multifocal pneumonia throughout the bilateral lungs; and a small right-sided pleural effusion (arrows) CT: computed tomography

The patient's serum sodium level remained within normal limits throughout the course of his hospital stay, which would prove significant later in his clinical course. Chest CT scan with angiography showed extensive mediastinal and hilar adenopathy, along with moderate to severe multifocal pulmonary infiltrates throughout the lungs (Figure 1). Infectious disease service was consulted, and they suspected pneumocystis pneumonia for which he was started on trimethoprim-sulfamethoxazole 160-800 mg (two tablets) every eight hours via nasogastric tube, along with an initial dose of dexamethasone 10 mg IV, followed by 6 mg IV daily. He was also started on anidulafungin with an initial dose of 200 mg IV followed by 100 mg IV daily, azithromycin 500 mg IV daily, piperacillin-tazobactam 3.375 mg IV every eight hours, and vancomycin IV, which was titrated per pharmacy protocol given a severe immunocompromised state. He was admitted to the ICU due to acute hypoxic respiratory failure for further evaluation and management.

**Figure 2:**
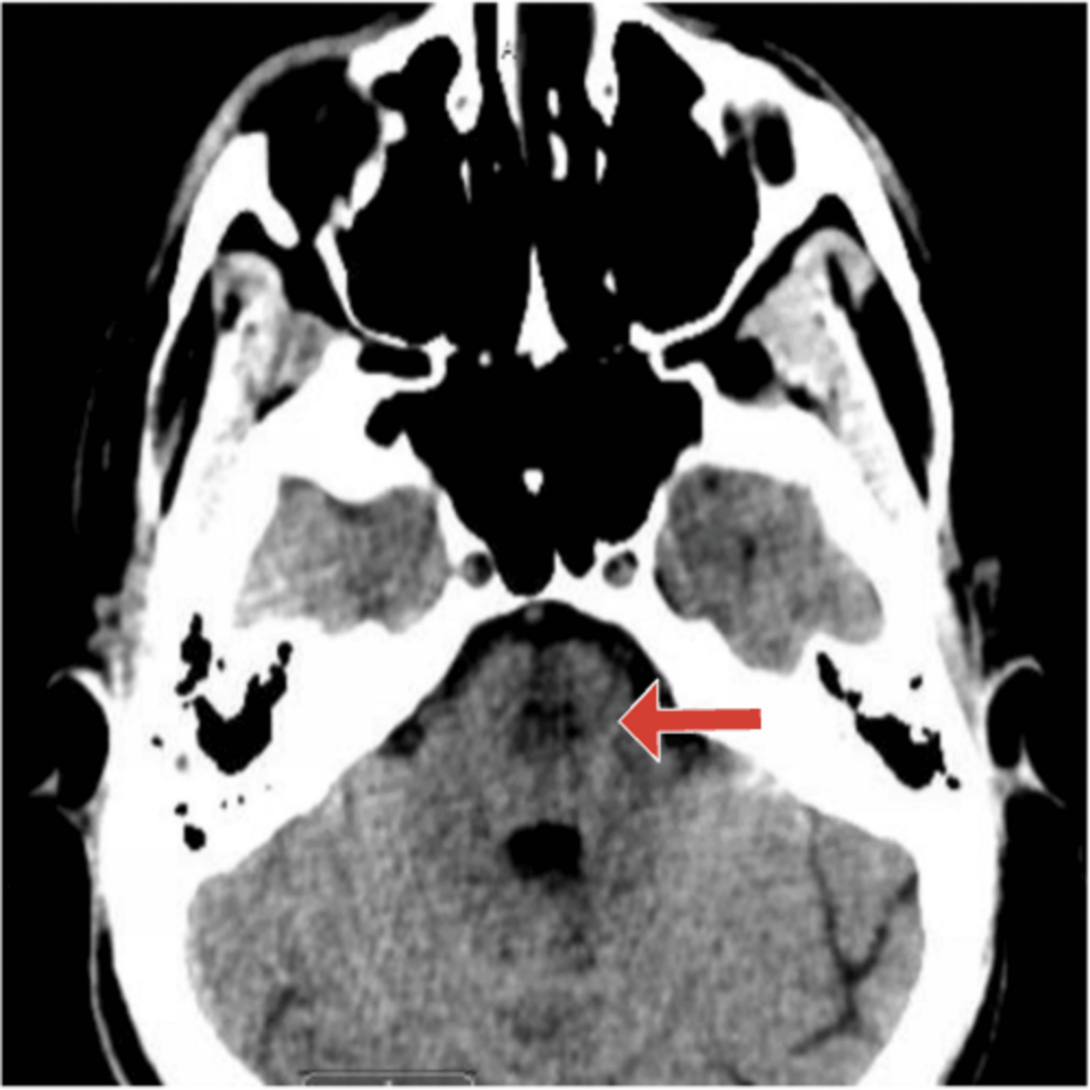
Head CT The image shows a hypodense area within the mid-pons (arrow), warranting further evaluation with MRI CT: computed tomography; MRI: magnetic resonance imaging

**Figure 3:**
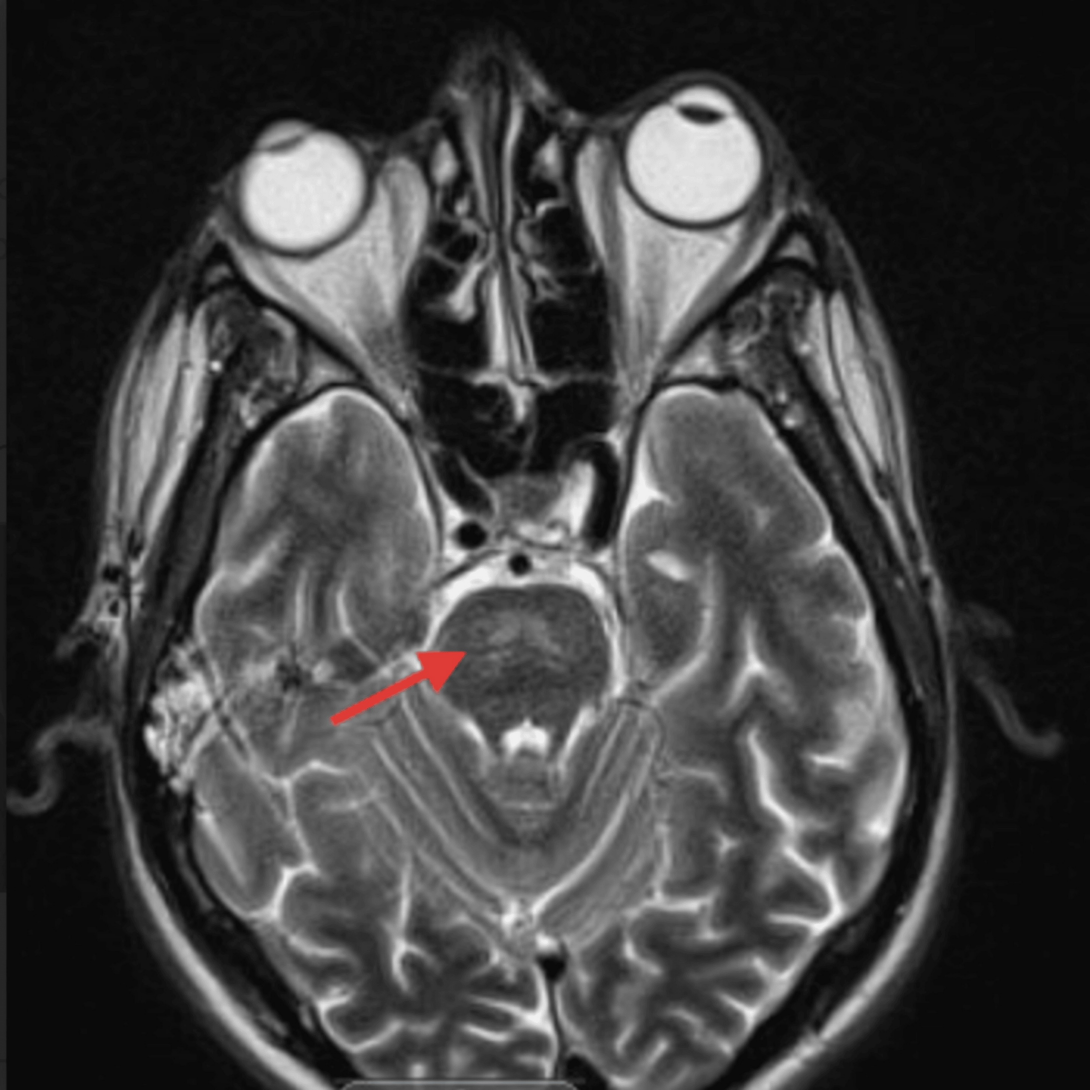
Brain MRI - image 1 The image shows T2-weighted FLAIR hyperintense lesions within the central pons, resembling an incomplete trident sign (arrow) MRI: magnetic resonance imaging: FLAIR: fluid-attenuated inversion recovery

**Figure 4:**
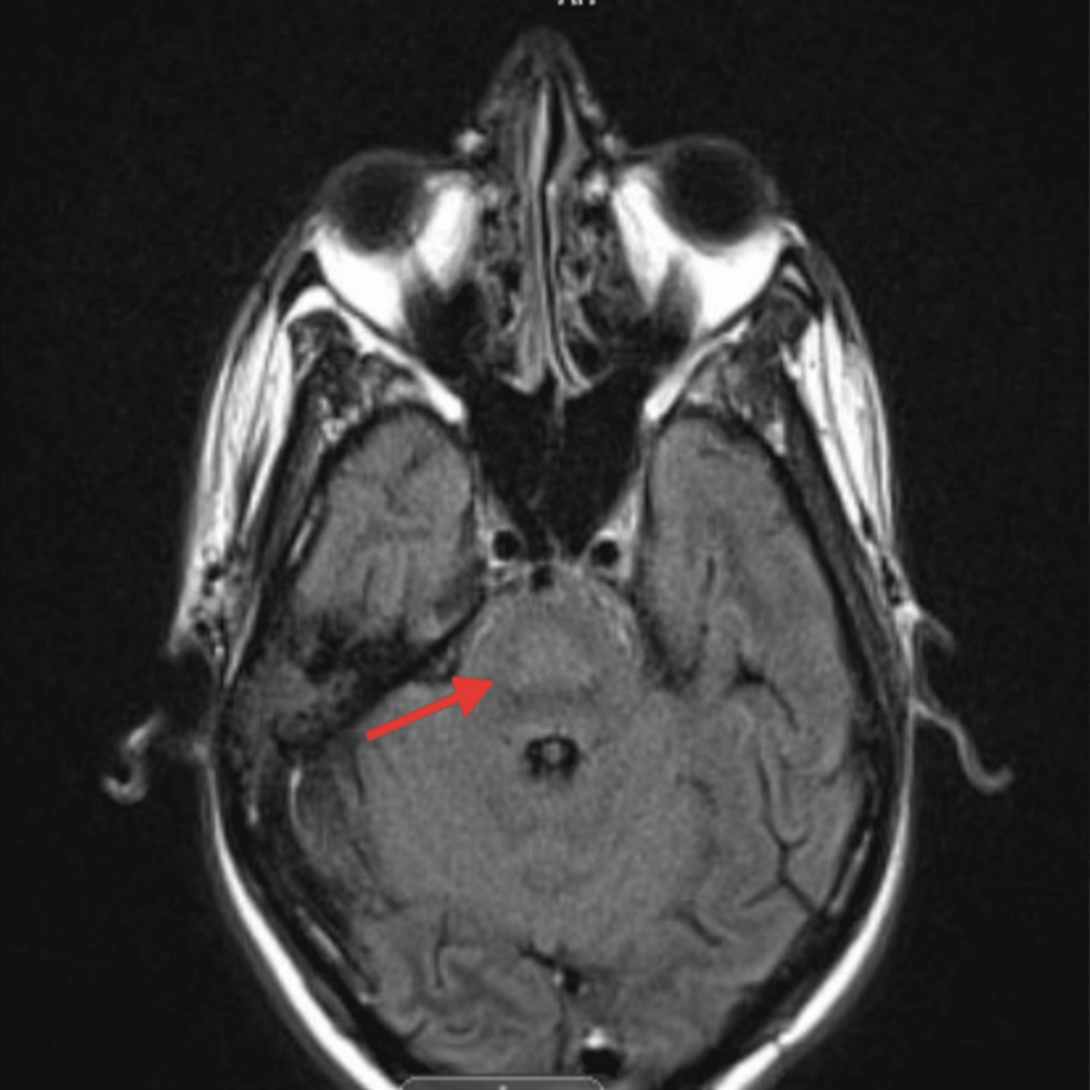
Brain MRI - image 2 The image shows diffusion restriction within the central pons, resembling an incomplete trident sign (arrow) MRI: magnetic resonance imaging

On ICU day three, the patient was noted to have new anisocoria, with the right pupil described as “enlarged and sluggish,” prompting an immediate neurologic evaluation. An initial head CT scan revealed a hypodense lesion in the mid-pons, raising concern for a structural brain abnormality (Figure 2). This finding prompted urgent MRI imaging, which demonstrated T2-FLAIR hyperintense lesions in the central pons, resembling an incomplete trident sign (Figures 3, 4).

A comprehensive neurologic examination was performed the next day without sedation for seven minutes, which revealed pupils measuring 2-3 mm bilaterally, positive corneal, gag, and cough reflexes, and brisk withdrawal to noxious stimuli in the extremities. We systematically explored potential causes for the patient's CPM, including review of recent and outpatient laboratory values, but found no evidence of sodium fluctuations or fluid administrations that might explain the pontine demyelination. Additional testing revealed positive serum CMV quantitative PCR with quantitative range assay of 200 to 1 million IU/mL, indicating an active viral infection. Lumbar puncture was also performed, which showed only mildly elevated opening pressure (34 cm H_2_O) and protein level (53 mg/dL), with otherwise unremarkable cerebrospinal fluid analysis (Table 2).

**Table 2:** CSF analysis following lumbar puncture CSF: cerebrospinal fluid; WBC: white blood cell; RBC: red blood cell; PCR: polymerase chain reaction; Ag: antigen; CMV: cytomegalovirus; HSV: herpes simplex virus; HHV: human herpes virus; VZV: varicella zoster virus; IgG: immunoglobulin G; IgM: immunoglobulin M; Ab: antibody; AFB: acid-fast bacillus

CSF analysis	Result	Normal values
Appearance	Clear	Clear
Color	Colorless	Colorless
Spun appearance	Colorless	Colorless
WBC	<3/u/L	0-10/uL
RBC	4/uL	0-5/uL
Mononuclear cells	0%	0%
Polymorphonuclear	100%	<2%
Glucose	65 mg/dL	40-70 mg/dL
Total protein	53 mg/dL	15-40 mg/dL
C. neoformans PCR	Not detected	Not detected
Cryptococcus Ag	Nonreactive	Nonreactive
CMV DNA (PCR)	Not detected	Not detected
Enterovirus (PCR)	Not detected	Not detected
E. coli (PCR)	Not detected	Not detected
H. influenza (PCR)	Not detected	Not detected
HSV 1 (PCR)	Not detected	Not detected
HHV 6 (PCR)	Not detected	Not detected
L. monocytogenes (PCR)	Not detected	Not detected
N. meningitidis (PCR)	Not detected	Not detected
Parechovirus (PCR)	Not detected	Not detected
S. agalactiae (PCR)	Not detected	Not detected
S. pneumoniae (PCR)	Not detected	Not detected
VZV (PCR)	Not detected	Not detected
West Nile IgG Ab	Negative	Negative
West Nile IgM	Negative	Negative
AFB culture	No fungus isolated after 4 weeks	No fungus isolated
CSF culture	No growth after 72 hours	No growth

The neurology team was consulted and reported that they did not find any serum sodium level <130 meq/L from any previous hospital/health care encounter. There was also no concern for multiple sclerosis, so oligoclonal band testing or additional imaging was not pursued. The oncology team was consulted to evaluate whether the patient's chemotherapy regimen might have contributed to his neurologic findings, but they determined that his clinical picture was not consistent with known neurotoxicity patterns associated with these chemotherapy agents. Given the patient's positive CMV PCR in the setting of severe immunosuppression, and despite negative CSF CMV results, the multidisciplinary team, including intensivists, infectious disease specialists, and neurologists, suspected CMV-related central nervous system involvement as a potential trigger for his CPM. Based on this clinical reasoning, we initiated treatment with valganciclovir 900 mg PO twice daily for a planned three-week course.

The patient was eventually weaned off the ventilator and was successfully extubated on ICU day seven. Subsequent neurologic examinations revealed a Glasgow Coma Scale (GCS) score of 14 (E=4, V=4, M=6) with intermittent confusion and bilateral pupils measuring 2-3 mm, while the rest of his cranial nerve function, motor and sensory systems, reflexes, coordination, and gait were normal. Meningeal signs were absent. Fundoscopic assessment was not undertaken owing to the lack of on-site ophthalmology services. Blood and endotracheal cultures did not grow any pathogenic microorganisms. Hence, sulfamethoxazole-trimethoprim, valgancyclovir, and dexamethasone were continued, while piperacillin-tazobactam, anidulafungin, and vancomycin were discontinued.

The patient was counseled extensively about the potential for delayed onset of neurologic symptoms and the critical importance of close monitoring and follow-up care. Unfortunately, the patient left against medical advice (AMA), stating that “he wanted to go outside.” He was determined by the house staff to have the capacity to make medical decisions for himself and later signed the AMA form despite discussions about the risks and benefits of continued hospitalization. Despite counseling efforts, the patient did not follow up regarding his medical conditions after discharge.

## Discussion

CPM, a subtype of ODS, represents a rare but potentially devastating demyelinating condition that primarily targets the central pons. Extrapontine regions may also be involved at times, including the basal ganglia, thalami, subcortical white matter, fornices, and cerebellum [1,3,5,6]. Clinical manifestations vary widely: some patients remain asymptomatic, while others may experience mild confusion, speech disturbances, or seizures. More severe presentations can include locked-in syndrome, spastic quadriparesis, pseudobulbar palsy, coma, or death [1,4-10]. In our patient, the observed neurological findings included mild confusion, slowed cognition, and anisocoria.

The incidence of ODS is rare in the general population. A nationwide study in Sweden reported an incidence of 0.611 per million person-years (95% CI: 0.490-0.754), including cases of CPM. The incidence increased over time, from 0.271 per million person-years between 1997 and 2001 to 0.945 per million person-years between 2007 and 2011, likely reflecting improved MRI availability and heightened clinical awareness [8]. However, determining the true incidence of CPM remains challenging. Many cases likely go undiagnosed, especially in patients whose neurological symptoms are attributed to other complex or critical conditions, where the differential diagnosis is broad and early imaging findings may be subtle [4,9]. MRI remains the most reliable diagnostic tool, typically demonstrating pathognomonic T2/FLAIR hyperintensity and diffusion restriction in the central pons, often with the characteristic trident or bat-wing appearance. However, as highlighted in a case series by Erkalayci et al., CPM can range from asymptomatic to fatal, and radiological pontine hyperintensities should prompt consideration of CPM among other differential diagnoses [7].

The pathophysiology of classic CPM involves osmotic shifts that damage oligodendrocytes and disrupt the blood-brain barrier, resulting in the characteristic symmetric demyelination pattern within the pontine region [4]. Remarkably, this process typically leads to myelin loss and spares both axon cylinders and neurons [10], which may explain why some patients can achieve meaningful recovery. For decades, clinicians have recognized CPM's strong association with rapid correction of symptomatic or chronic hyponatremia. This relationship is so well-established that many practitioners automatically look for recent sodium fluctuations when encountering pontine lesions on neuroimaging [7].

Interestingly, a growing body of evidence suggests that not all CPM cases involve electrolyte abnormalities [8,9]. A review on CPM concluded that CPM is frequently associated with other underlying conditions, including chronic alcoholism, malnutrition, uremia, dialysis-related complications, liver dysfunction, dehydration, diabetes mellitus, electrolyte imbalances beyond sodium, and metabolic disorders such as hypoalbuminemia [1,3,5,7]. Reports of normonatremic CPM have also emerged, highlighting alternative pathogenic mechanisms, particularly in the context of severe systemic stress or infection. These observations have significant implications for the clinical assessment and management of affected patients.

Several case reports have suggested that either HIV or AIDS can be the primary cause or a predisposing factor of ODS. These cases either had opportunistic infections, lymphoma, or ongoing chemotherapy leading to metabolic derangements [11-16]. CMV infection is well recognized as a significant opportunistic pathogen in immunocompromised individuals, particularly in those with HIV/AIDS or undergoing chemotherapy for malignancies or autoimmune disorders. CMV's neurotropism is well-documented, causing at least five distinct neurological syndromes in patients with AIDS, including myelitis, polyradiculopathy, encephalitis with dementia, ventriculoencephalitis, and mononeuritis multiplex [17]. However, CMV and HIV’s potential role in triggering CPM remains poorly understood, with only a few cases documented in the medical literature. One such report describes a 10-month-old infant with CMV hepatitis [5]. In this report, it is proposed that CMV infection disrupts the hepatic function and electrolyte balance, leading to a cascade of metabolic and osmotic insults resulting in cellular dehydration, astrocyte and oligodendrocyte injury, blood-brain barrier disruption, and ultimately demyelination [5].

In our patient, although CSF analysis for CMV was negative, it is important to note that lumbar puncture has a reported sensitivity of 82% and specificity of 99% for detecting CNS infection. Given the positive serum CMV PCR test, we considered the negative CSF result a potential false negative based on the overall clinical picture, including his signs and symptoms, CD4 count being nil, and a positive serum CMV DNA indicating an active CMV infection. Further diagnostic confirmation, such as repeat CSF PCR or ophthalmologic examination, was not possible because the patient left AMA. Thus, it may be hypothesized that immunocompromised patients with AIDS complicated by an opportunistic infection like CMV and cancer patients receiving chemotherapy may be more susceptible to metabolic disturbances resulting in hypoalbuminemia due to increased serum cytokines and metabolites associated with their complex medical conditions [15].

Although causation remains uncertain, a 2002 case report by Tarhan et al. described a patient who achieved a good clinical recovery despite no follow-up MRI [5]. One possible hypothesis is that early treatment of these infections can mitigate metabolic disturbances, leading to a good overall clinical picture by reducing systemic stress, preventing further neurologic deterioration, and optimizing the environment for neurologic recovery. The outcome can range from death or permanent disability to near-complete recovery [1,8]. But in a nationwide study involving 83 patients, 50% of patients with CPM were later functionally independent [1,8]. This highlights the importance of prompt treatment of associated conditions like HIV/AIDS and CMV infections to prevent further complications. However, supportive care and neurorehabilitation remain the cornerstone of managing CPM itself.

## Conclusions

This report illustrates that CMV viremia in profound immunosuppression can coincide with CPM-like MRI findings despite the absence of serum sodium abnormalities, emphasizing the importance of heightened awareness when encountering severely immunocompromised patients presenting with unexpected MRI findings. Suspected CMV-associated CPM in immunocompromised patients has been sparsely reported in the literature, but when recognized early and treated appropriately, patients have shown favorable outcomes. This highlights the importance of early recognition and timely intervention, as prompt diagnosis and initiation of antiviral therapy can significantly improve overall prognosis.
